# Sex differences in cardiac remodeling post myocardial infarction with acute cigarette smoking

**DOI:** 10.1186/s13293-022-00446-y

**Published:** 2022-07-07

**Authors:** Abdullah Kaplan, Emna Abidi, Reine Diab, Rana Ghali, Hiam Al-Awassi, George W. Booz, Fouad A. Zouein

**Affiliations:** 1 Department of Pharmacology and Toxicology, Medical Center, American University of Beirut, Faculty of Medicine, Riad El-Solh, Beirut, 1107 2020 Lebanon; 2 Department of Cardiology, Kemer Public Hospital, Hastane Cd. No: 9, 07980 Kemer, Antalya Turkey; 3 The Cardiovascular, Renal, and Metabolic Diseases Research Center of Excellence, American University of Beirut Medical Center, Riad El-Solh, Beirut, Lebanon; 4 Department of Pharmacy, Cleveland Clinic Abu Dhabi, Abu Dhabi, UAE; 5 Department of Pharmacology and Toxicology, School of Medicine, University of Mississippi Medical Center, Jackson, MS 39216-4500 USA; 6 Department of Signaling and Cardiovascular Pathophysiology, UMR-S 1180, Inserm, Université Paris-Saclay, Paris, France

**Keywords:** Left ventricular remodeling, Right ventricular remodeling, Cigarette smoke, Sex differences, Cardiac fibrosis, Inflammation

## Abstract

**Background:**

Whether cigarette smoking affects the heart post-myocardial infarction (MI) in a sex-dependent way remains controversial. Using a mouse model, we investigated cardiac remodeling under the influence of acute cigarette smoke (CS) exposure following ischemic injury in both sexes.

**Methods:**

Ten cigarettes were smoked twice daily for 2 weeks followed by MI and then 1 additional week post permanent LAD ligation. Cardiac function, histology, and infarct size were assessed, and inflammatory markers quantified by RT–PCR. Statistical comparisons were performed using an unpaired *t* test or ANOVA followed by Tukey post hoc test.

**Results:**

We observed that cigarette smoking exacerbated both left and right ventricular remodeling only in males at an early stage of post-MI. Females did not display a significant structural and/or functional alteration within 7 days of cardiac remodeling post-MI upon CS exposure. Worsened right ventricular remodeling in males was independent of pulmonary congestion. CS-exposed males exhibited enhanced increases in left ventricular end systolic and diastolic volumes, as well as reductions in ejection fraction and fractional area changes of left ventricular base. At day 7, infarct size was increased by cigarette smoking in males only, which was accompanied by enhanced collagen deposition in both the infarcted and peri-infarcted areas. Both IL-6 and TNF-α mRNA expression significantly increased in CS-exposed MI male group only at day 7 post-MI suggestive of prolonged inflammation.

**Conclusions:**

These findings indicate that CS exposure worsens the progression of cardiac remodeling post-MI in male sex in a significant manner compared to female sex at least at early stages.

## Introduction

The prevalence of cardiovascular disease (CVD) is steadily rising and is expected to increase by a minimum of 10% over the next 20 years. Worldwide, yearly deaths of 23.6 million are projected for CVD by 2030 [1, 2]. In addition, costs of myocardial infarction (MI) to health care will increase by threefold over the next two decades [3]. Left ventricular (LV) remodeling is an alteration in size, shape, and function of the heart due to genomic, molecular, cellular, and extracellular changes resulting from cardiac injury or stress [4, 5]. Different pathological and physiological stimuli are responsible for cardiac remodeling. Ischemic injury due to coronary artery disease is the most common pathological cause of cardiac remodeling [6]. Ventricular hypertrophy and/or dilatation are main structural changes that reflect a pathological response of the heart, accompanied by molecular and cellular changes that functionally translate into diastolic and/or systolic dysfunction [7]. With acute coronary artery disease, heart failure incidence is common and the cumulative 1 year heart failure rate among patients who had transmural MI is 23.4% [8].

Tobacco smoking is one of the major risk factor for coronary heart disease [9, 10]. Beside a direct toxic effect on myocytes (smoking cardiomyopathy), smoking is also an indirect contributor of cardiac pathology by fueling comorbidities, such as atherosclerotic syndromes and hypertension that also damage and remodel the heart [7]. Estimates of global mortality due to smoking worldwide indicate that a higher number of smokers die from heart disease than from respiratory disease or all forms of cancer combined [11]. In fact, reported cardiovascular effects of smoking are multiple and diverse ranging from the effect of traditional CVD risk factors, such as high blood pressure (BP), dyslipidemia, insulin resistance, to subclinical factors, such as coronary artery calcification, increased carotid intima–media thickness, and thrombosis development, affecting autonomic nervous system balance, and even cardiac electrophysiology, performance, and mass. Biomarkers of cardiovascular harm are also increased by smoking as a main independent CVD risk factor that triggers vascular injury, stiffness, and inflammation [12].

The World Health Organization (WHO) describes the tobacco epidemic as one of the largest public health threats the world has faced. More than 7 million people die due to smoking-associated diseases per year [11]. More than 6 million of those deaths are attributed to direct tobacco use, while around 890,000 deaths are the result of non-smokers being exposed to second-hand smoke (http://www.who.int/mediacentre/factsheets/fs339/en/). Although consumption of tobacco products has decreased in some high- and upper middle-income countries, it has markedly increased in developing regions [13, 14]. In parallel, the total number of deaths attributed to smoking has decreased in many rich countries, whereas it has increased in most low- and middle-income countries in recent decades due to population growth and ageing in these countries (https://ourworldindata.org/smoking#smoking-is-one-of-the-leading-risks-for-early-death).

A positive correlation between tobacco smoking and cardiovascular risk has been described for both men and women with smoking still representing a major contributor to the top cardiovascular disease risk factors in 2022 [15]. Young women who smoke have an increased risk for sudden death with MI being the most strongly associated. In men who smoke, the risk of developing MI is reported to be about 5 times higher compared to women and increases with the number of cigarettes smoked. This sex-based difference has been largely attributed to a beneficial protective role of female hormones on the cardiovascular system [16]. Contradictory reports have suggested that smoking women are more prone to develop worse adverse health outcome than smoking men [17]. This has been attributed to the combination of many factors. Genetic and biological factors, hormonal factors, socioeconomic factors, occupational exposure, work stress, personal lifestyle, and second-hand smoke exposure may be responsible for adverse health outcomes in women [18]. This debate occurs along with the rising epidemic of smoking among women, while the prevalence of smoking among men is in slow decline [17].

With respect to coronary heart disease, a meta-analysis reported that women who smoke have a 25% greater relative risk than their male counterparts independent of other cardiovascular risk factors [19]. The risks of smoking between sexes are clinically well documented; however, the direct impact of cigarette smoke (CS) on cardiac remodeling lacks a fundamental explanation. Female and male hearts share similar anatomic and physiologic features with slight exceptions, such as smaller LV cavity and higher heart rate in females [20]. Taking into consideration differences in genetic, biological, hormonal, and socioeconomic factors, it is not surprising to expect different response of the female heart to the same extent of injury as males, and most of the few studies on sex differences in cardiac remodeling were studied under influence of several stressors including ischemic damage or volume and pressure overload [20–25]. Results of most studies favored a benefit of female sex [25]. Understanding sex differences in response to cardiac injury will allow us to minimize bias in management and treatment of men and women. In addition, the exploration of underlying mechanisms of different responses in the two sexes might open up new insights into drug development to attenuate adverse cardiac remodeling. In the present study, we aimed to investigate cardiac remodeling under the influence of acute smoke exposure following ischemic injury in both sexes.

## Materials and methods

### Animals

This study was approved by the Institutional Animal Care and Use Committee of the American University of Beirut (AUB). Male and Female C57BL/6 J mice, 5 months in age and, respectively, 31 and 24 g in average weight were used. Animals were purchased from Charles River Laboratories (Wilmington, MA, USA) and housed at the AUB animal care facility under pathogen-free conditions with constant temperature and humidity control.

### Experimental protocol

All surgical procedures were performed under deep anesthesia. Mice were allocated into six groups as follows: control female/control male (CF/CM), control groups that neither underwent surgery nor was exposed to CS; MI female/MI male (MIF/MIM), both groups underwent permanent LAD ligation via microscopic surgery and were sacrificed 1 week after surgery; and smoke MI female/smoke MI male (SMIF/SMIM), mice in this group were subjected to 2 weeks of CS just before MI, after which mice were exposed again to 1 more week of CS.

### Echocardiography

Transthoracic echocardiography was performed using the Vevo 2100™ High-Resolution Imaging System (Visual Sonics, Toronto, Canada). Data were collected at baseline, the day before surgery, as well as days 1 and 7 after surgery. For image acquisition, mice were anesthetized with 2% isoflurane in an oxygen mix chamber and placed on an electrical heated platform. B-mode images of the left ventricle were acquired from the parasternal long axis view in supine position. LV end diastolic (LVEDV) and systolic volumes (LVESV) were provided by the machine using the LV wall tracing tool. LV ejection fraction (EF %) was calculated from B-mode image as previously described [26]. In addition, images of base, midsection, and apex were acquired from short axes view. The images of the base acquired at day 1 post MI was used for calculation of fractional area change. Endocardial border was traced manually at end-diastole and at end-systole, and subsequently percentage of area change was calculated. Body temperature, heart rate, and respiratory rate were continuously monitored throughout the procedure via Indus Mouse Monitor Heated Surgical Platform and the depth of anesthesia was adjusted accordingly.

### Cigarette smoking exposure

Mice were exposed to CS using an exposure apparatus with single animal modules (ONARES, CH Technologies, NJ, USA) as previously described [27–29]. Mice were familiarized with the apparatus over the 7 days prior by daily exposures without CS. The CS apparatus includes a smoke generator with a mixing/conditioning chamber and a “nose only” rodent exposure carousel. This system allows for exposure to mainstream smoke from a cigarette in conscious, restrained rodents and has been extensively used to study smoking-related diseases (https://chtechusa.com/publications-byproducts-scsm.php). After arrival in the smoking exposure room, mice were allowed to acclimate for 15–30 min before initiating the CS protocol. Ten cigarettes were smoked twice daily (7 days/week) either for 3 continuous weeks for the smoking only male and female groups, or for 2 weeks followed by myocardial infarction (MI) and then 1 additional week post permanent LAD ligation. Cigarettes were placed into the cigarette puffer, and a peristaltic pump was used to generate puffs at a frequency of one puff/min, duration of 2.5 s, and puff volumes of 5 mL generated from 3R4F cigarettes (University of Kentucky, Lexington, KY, USA). 3R4F are scientifically prepared cigarettes concentrated with toxins and chemical rendering the study timeline suitable to observe the effects of smoking on mice. Animals received two 60 min CS sessions per day allowing a total particular matter concentration of about 100 g/cm^3^/mouse/session.

### Blood pressure measurements

Blood pressure (BP) was measured non-invasively using tail cuff, volume pressure recording sensor technology and CODA high-throughput monitor, (Kent Scientific, Torrington, CT, USA). Measurements were performed at baseline and after 2 weeks of CS exposure to assess the impact of CS on BP in both CS exposed groups. Real-time measurements of systolic, diastolic, and mean arterial blood pressure were recorded. Each session consisted of 5 acclimatization cycles followed by 15 BP measurement cycles. On the data collection day, 2 sessions of 15 BP measurements were obtained; any irregular recordings noted as false recordings by the system were excluded. Briefly, mice were trained for 7 days by measuring BP daily, after which BP recordings were made and a set was accepted if the computer identified 50% successful readings. The average from one session was used for systolic BP, diastolic BP, and mean BP in each individual mouse.

### Myocardial infarction surgery

MI was induced by permanent ligation of the left anterior descending coronary (LAD) artery. Briefly, the procedure is as follows: mice were anesthetized with isoflurane (2–3% in oxygen) and placed on a heating pad to prevent hypothermia. Before surgery, mice were given tramadol as analgesic. First, a skin excision was performed to expose the trachea followed by orotracheal intubation with the tube connected to a mini-ventilator (Harvard Apparatus, Holliston, MA, USA). Second, animals were placed in lateral supine position and the skin on the left hemithorax was excised. Following the retraction of pectoralis and intercostal muscles, the pericardium was dissected and the LAD artery exposed. MI was induced by permanent ligation of the LAD coronary artery 1–3 mm from the tip of the left atria. Occlusion of LAD was confirmed by the appearance of a pale color in the anterior wall of the left ventricle and ST elevation on the electrocardiogram using the Indus Mouse Monitor system. The thorax was then closed and mice were kept warm until they freely groomed and were monitored closely and daily until recovery.

### Necropsy

At day 7 post-MI, mice were sacrificed under deep anesthesia; 2–3% isoflurane was constantly delivered using an oxygen-based isoflurane vaporizer. Anesthetic depth was assessed by toe pinch before starting the necropsy procedure. The heart was harvested, and the left ventricle separated from the right ventricular (RV) cavity under microscope. Both ventricles were weighed separately Lungs were harvested and weighed as wet lung mass before being placed in an incubator for 24 h to dry. To determine fluid accumulation in the lung post-MI, wet lung weight was subtracted from dry lung mass and divided by wet lung mass. The left ventricle was sliced into sections: base, mid-section, and apex. The base section was processed for RNA extraction. The mid-section was processed for histological analysis.

### Infarct size measurements

At day 1 post MI, echocardiographic images from base, mid and apex of left ventricle were utilized for evaluation of infarct size. As previously reported [28, 30], infarct region was identified from each of the short axis views by visual assessment of wall thickening and wall motion. Thereafter, length of infarct was manually traced along the endocardial border at end-diastole. The infarct size was estimated as the percentage of the total infarct length to the total LV endocardial circumferential length. Due to lack of infarct on base, mid and apex of LV were taken into calculation. At study completion, infarct size was assessed using 2,3,5-triphenyltetrazolium chloride (TTC) (Sigma-Aldrich, St. Louis, MO, USA) stain. Each left ventricle was sliced horizontally into 3 slices. RV and LV slices were incubated in 1% TTC for 15 min at 37 °C. The infarcted region was defined as the unstained section following incubation with TTC. ImageJ software (National Institutes of Health, Bethesda, MD, USA) was used for infarct size measurement. The whole LV area along with the unstained region was traced and the measurement was done automatically by the program in pixels. The infarct area was expressed as a percentage of the left ventricle. As for RV area, the borders of the RV were traced, and measurement was done automatically. No infarcted area was detected on the RV tissue.

### Masson’s trichrome staining

For myocardial fibrosis and collagen deposition, the slides of the left ventricle’s mid-section were stained with Masson’s trichrome. Collagen volume fractions (CVFs) and fibrosis were measured using ImageJ software (https://imagej.nih.gov/ij/). Briefly, after dewaxing and hydration steps, and according to manufacturer’s protocol (Abcam, Connective Tissue Stain, #ab150686) tissue slides were soaked in Bouin solution for 1 h at 56 °C, washed in running tap water, and rinsed in distilled water. A second washing step was done after 10 min of incubation in hematoxylin. Slides were stained in biebrich scarlet acid fuchsin for 10 min. After washing, sections were differentiated in phosphomolybdic–phosphotungstic acid solution for 10 min, transferred to aniline blue solution, stained for 5 min and mounted and observed using light microscopy at 400 × magnification. Images from infarcted and peri-infarcted areas were separately taken. Seven images per area and slide were acquired with an *n* = 6.

### RNA extraction and real time–quantitative polymerase chain reaction (RT–qPCR)

Snap frozen tissue (base section of the left ventricle) was used for RNA extraction. Briefly, tissues were grounded in liquid nitrogen with mortar and pestle and total RNA isolated using TRIzol according to the manufacturer’s instructions (ThermoFisher Scientific, Grand Island, NY, USA). RNA quantification was done with a NanoDrop^®^ ND-1000 and purity assessed using the 260–280 nm absorbance ratio. To remove contaminating DNA, RNAs were treated with deoxyribonuclease I (ThermoFisher Scientific). cDNA was synthesized using the Revert Aid 1st Strand cDNA synthesis kit (ThermoFisher), followed by real-time PCR in a CFX96 real-time PCR system with SYBR^®^ Green PCR Master Mix (Bio-Rad, Hercules, CA, USA). cDNA was loaded in duplicate with each forward and reverse primers of the gene of interest. HPRT expression was used to normalize gene expression between different samples. Negative control (RNA-free water) was used to check for nonspecific amplification. Fold-expressions were normalized relative to the control, and were calculated and plotted using Bio-Rad CFX Manager to compare differential gene expressions. The following primers were obtained from Macrogen (Seoul, South Korea):

**Table:** 

TNF-α	
Forward	5′-TGT GCT CAG AGC TTT CAA CAA-3′
Reverse	5′-CTT GAT GGT GGT GCA TGA GA-3′
IL-6	
Forward	5′-CAA CGA TGA TGC ACT TGC AGA-3′
Reverse	5′-GTG ACT CCA GCT TAT CTC TTG GT-3′
IL1-β	
Forward	5′-TGG TGT GTG ACG TTC CCA TT-3′
Reverse	5′-TGT CGT TGC TTG GTT CTC CT-3′

### Statistical analysis

Results were expressed as mean ± the standard error of the mean (SEM). Statistical comparisons were performed using an unpaired *t* test or for multiple comparison, ANOVA followed by Tukey post hoc test was performed. *p* ≤ 0.05, *p* ≤ 0.01, and *p* ≤ 0.001 (*, **, ***, respectively) were considered significant. GraphPad Prism software was used to perform statistical analysis.

## Results

### CS exposure did not alter arterial blood pressure prior to MI in either sex

**Fig. 1:**
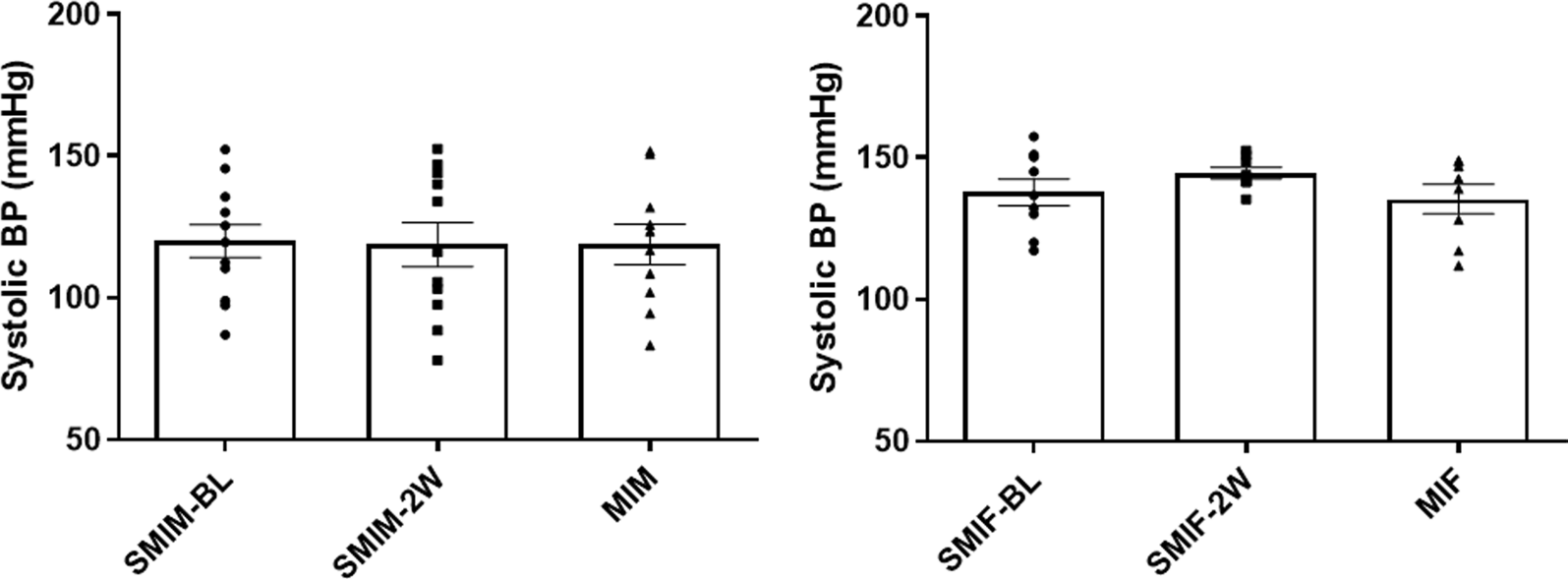
Blood pressure measurement after 2 weeks of cigarette smoke (CS) exposure prior to surgery. After 2 weeks of CS exposure, systolic blood pressure did not show a significant alteration from baseline in either sex compared to their control counterparts MIF: MI female group (*n* = 8); MIM: MI male group (*n* = 10); SMIM-BL: smoking Male group before starting CS exposure (*n* = 12); SMIF-BL: smoking Female group before starting CS exposure (*n* = 9); SMIM-2 W: 2 weeks of CS exposure for smoking male group (*n* = 11); SMIF-2 W: 2 weeks of CS exposure for smoking female group (*n* = 8)

Table 1 shows the echocardiographic and blood pressure parameters at baseline for male and female mice with or without smoking. Both male and female CS exposed groups did not show a significant alteration in blood pressure following 2 weeks of CS exposure and prior to MI induction compared to their age and gender matched control counterparts (Fig. 1).

**Table 1:** Echocardiographic and blood pressure measurements the day before LAD ligation

Group	Heart Rate (bpm)	Area; systole (mm^2^)	Area; diastole (mm^2^)	Volume; Systole (μL)	Volume; diastole (μL)	Stroke Volume (μL)	Ejection fraction (%)	Systolic Blood Pressure (mmHg)
MIF	437.63 ± 20.46	0.79 ± 0.04	1.35 ± 0.05	1.46 ± 0.10	3.66 ± 0.20	2.19 ± 0.13	60.09 ± 1.51	144.48 ± 2.04
SMIF	436.31 ± 19.13	0.83 ± 0.04	1.39 ± 0.05	1.69 ± 0.12	3.94 ± 0.17	2.24 ± 0.10	57.19 ± 1.94	135.35 ± 5.19
MIM	460.76 ± 27.18	1.03 ± 0.04	1.64 ± 0.04	2.28 ± 0.15	4.95 ± 0.19	2.66 ± 0.10	53.95 ± 1.87	118.80 ± 9.22
SMIM	452.97 ± 18.84	1.04 ± 0.04	1.69 ± 0.06	2.30 ± 0.12	5.22 ± 0.27	2.92 ± 0.18	55.81 ± 1.23	118.76 ± 10.57

Values are shown as mean ± SEM

### CS exposure worsens left ventricular remodeling in males post-MI

**Fig. 2:**
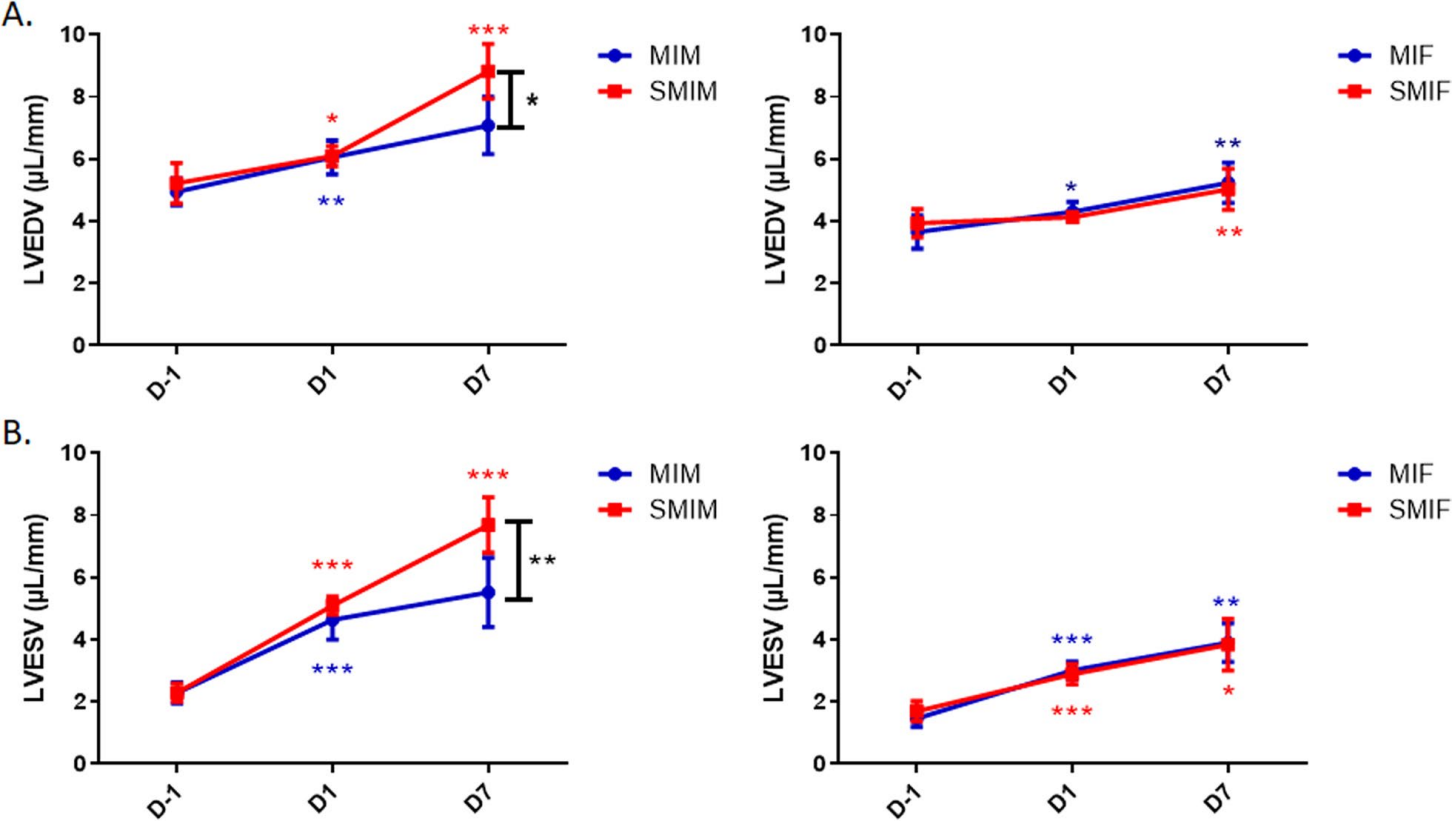
Effects of myocardial infarction (MI) and cigarette smoke (CS) on left ventricular volumes in males and females. Following MI surgery, both sexes displayed progressive LV dilatation through 7 days. **A** Left ventricular end-diastolic volume (LVEDV) of the CS-exposed and CS-naïve groups were compared at the day before surgery (BL/2 W), day 1, and day 7 post-MI. While LVEDV in both female groups was comparable at all timepoints, CS-exposed MI male group differed significantly from CS-naive MI male group at day 7. **B** Left ventricular end-systolic volume (LVESV) of the CS-exposed and CS-naive groups were compared at the day before surgery (day − 1), day 1, and day 7 post-MI. While LVESV in both female groups was comparable at all timepoints of assessment, CS-exposed MI male group differed significantly from CS-naïve MI males at day 7. *N* = 8 (MIF), 6 (MIM), 8 (SMIF), and 6 (SMIM). MI: myocardial Infarction; MIF: MI females group; MIM: MI male group; SMIF: smoking MI females group; SMIM: smoking MI males group; D-1: baseline the day before LAD ligation for MI group or the day before LAD ligation but after 2 weeks of CS exposure for smoking MI group; D1: day 1 after MI; D7: day 7 after MI, LV: left ventricular. *p* ≤ 0.05 (*), *p* ≤ 0.01 (**), and *p* ≤ 0.001 (***); ANOVA

**Fig. 3:**
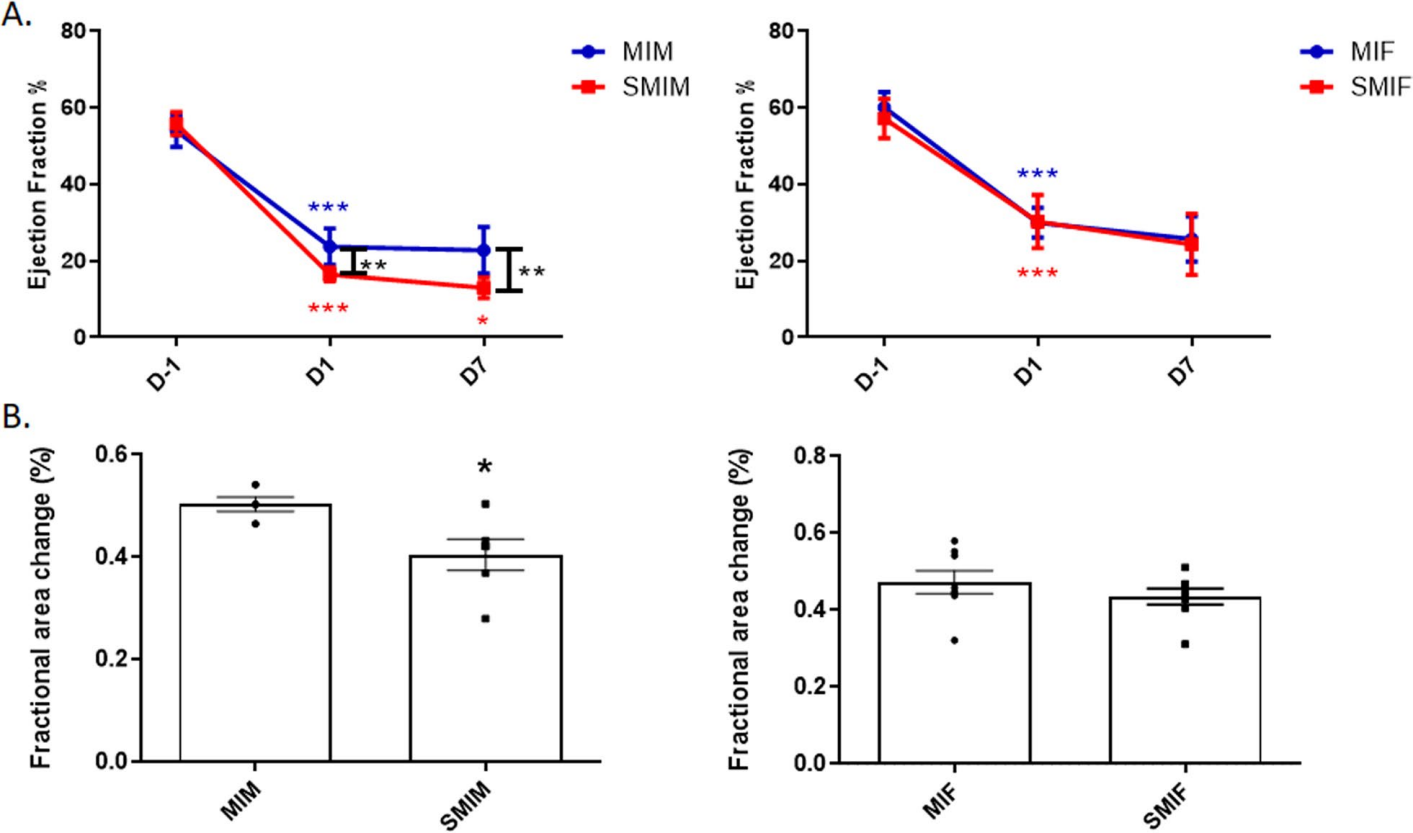
Effects of myocardial infarction (MI) and cigarette smoke (CS) on fractional area change and ejection fraction (EF) of LV in males and females. Following MI surgery, left ventricular systolic function progressively deteriorated during 7 days of follow-up. **A** Left ventricular EF of the CS-exposed and CS-naive groups were compared at the day before surgery (day − 1), day 1 and day 7 post-MI. While EF in both female groups was comparable at all timepoints of assessment, CS-exposed MI male group differed significantly from CS-naive MI males at day 1 and day 7 post-MI. **B** Fractional area change of LV base was found significantly reduced in CS-exposed MI male group in comparison with CS-naïve MI males at day 1 post-MI. No significant difference was found between female groups. *N* = 8 (MIF), 6 (MIM), 8 (SMIF), and 6 (SMIM). MI: myocardial Infarction; MIF: MI females group; MIM: MI male group; SMIF: smoking MI females group; SMIM: smoking MI males group; D-1: baseline the day before LAD ligation for MI group or the day before LAD ligation but after 2 weeks of CS exposure for smoking MI group; D1: day 1 after MI; D7: day 7 after MI, LV: left ventricular. *p* ≤ 0.05 (*), *p* ≤ 0.01 (**), and *p* ≤ 0.001 (***); ANOVA (**A**) and *t* test (**B**)

After 2 weeks of CS exposure, no group showed a significant change in LVEDV and LVESV nor EF compared to their CS-naïve counterparts (Figs. 2 and 3). Although the four groups of mice with comparable infarct size manifested no significant differences with smoking in LVEDV and LVESV by day 1 post-MI, EF was significantly depressed at day 1 in the CS-exposed MI male group (SMIM) compared to their nonsmoking counterpart (MIM), 16.43 ± 0.74 vs. 23.71 ± 1.94, *p* ≤ 0.01 (Fig. 3A).

**Fig. 4:**
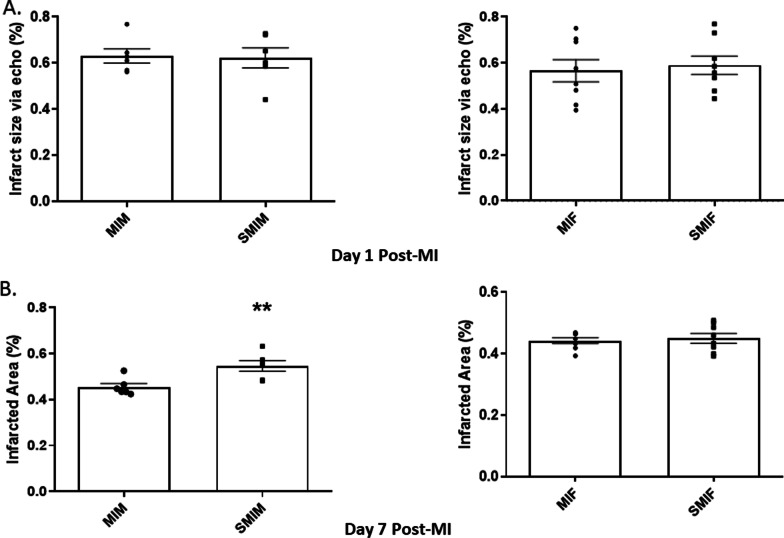
Comparison of infarct sizes among the groups. **A** No group showed significant difference with regard to echocardiographic measurements of infarct size at day 1 post-MI. **B** Only CS-exposed MI male group showed significantly larger infarcted area in the heart tissue at day 7 post-MI compared to their CS-naïve counterpart. *N* = 8 (MIF), 6 (MIM), 8 (SMIF), and 6 (SMIM). *MI* myocardial infarction, *MIF* MI female group, *MIM* MI male group, *SMIF* smoking MI female group, *SMIM* smoking MI male group. *p* ≤ 0.01 (**); *t* test

In line with what was observed with EF, CS-exposed MI male mice showed significantly lower fractional area at day 1 post-MI as compared to CS-naïve MI male mice, 0.40 ± 0.03 vs. 0.50 ± 0.01 *p* ≤ 0.05 (Fig. 3B). Smoke exposure did not result in a significant alteration in fractional area change in female mice, 0.43 ± 0.02 SMIF vs. 0.47 ± 0.03 MIF (Fig. 3B). At day 7 post-MI, the SMIM group had significantly larger LVESV (7.69 ± 0.36 vs. 5.52 ± 0.46, *p* ≤ 0.05) and LVEDV (8.82 ± 0.36 vs. 7.08 ± 0.38, *p* ≤ 0.01) (Fig. 2A, B) along with lower EF (12.97 ± 1.08 vs. 22.8 ± 2.48, *p* ≤ 0.01) compared to the MIM counterpart (Fig. 3A). MI reduced EF in female mice at day 1 (29.99 ± 1.37 MIF and 30.22 ± 2.47 SMIF) with no further significant decrease at day 7 nor with CS (25.69 ± 2.07 MIF and 24.29 ± 2.84 SMIF) (Fig. 3A); however, both female groups showed moderate and equivalent increases from day 1 in LVESV (3.01 ± 0.10 to 3.90 ± 0.22 MIF and 2.88 ± 0.12 to 3.84 ± 0.30 SMIF) and LVEDV (4.31 ± 0.11 to 5.24 ± 0.23 MIF and 4.13 ± 0.05 to 5.03 ± 0.23 SMIF) at day 7 post-MI, with LV adverse remodeling in progress through 7 days (Figs. 2 and 3). Although echocardiographic measurements of the infract size at day 1 were comparable between all groups (0.63 ± 0.03 MIM, 0.62 ± 0.04 SMIM, 0.57 ± 0.05 MIF 0.59 ± 0.04 SMIF) (Fig. 4A), differences were evidenced at day 7 on harvested heart tissue as LV remodeling progressed. The SMIM group exhibited larger infarct size than the MIM male group, 0.55 ± 0.02 vs. 0.46 ± 0.01, *p* ≤ 0.01 (Fig. 4B). No significant difference was found among female groups, 0.45 ± 0.01 SMIF vs. 0.44 ± 0.01 MIF.

### CS exposure enhances left ventricular mass in MI males

**Fig. 5:**
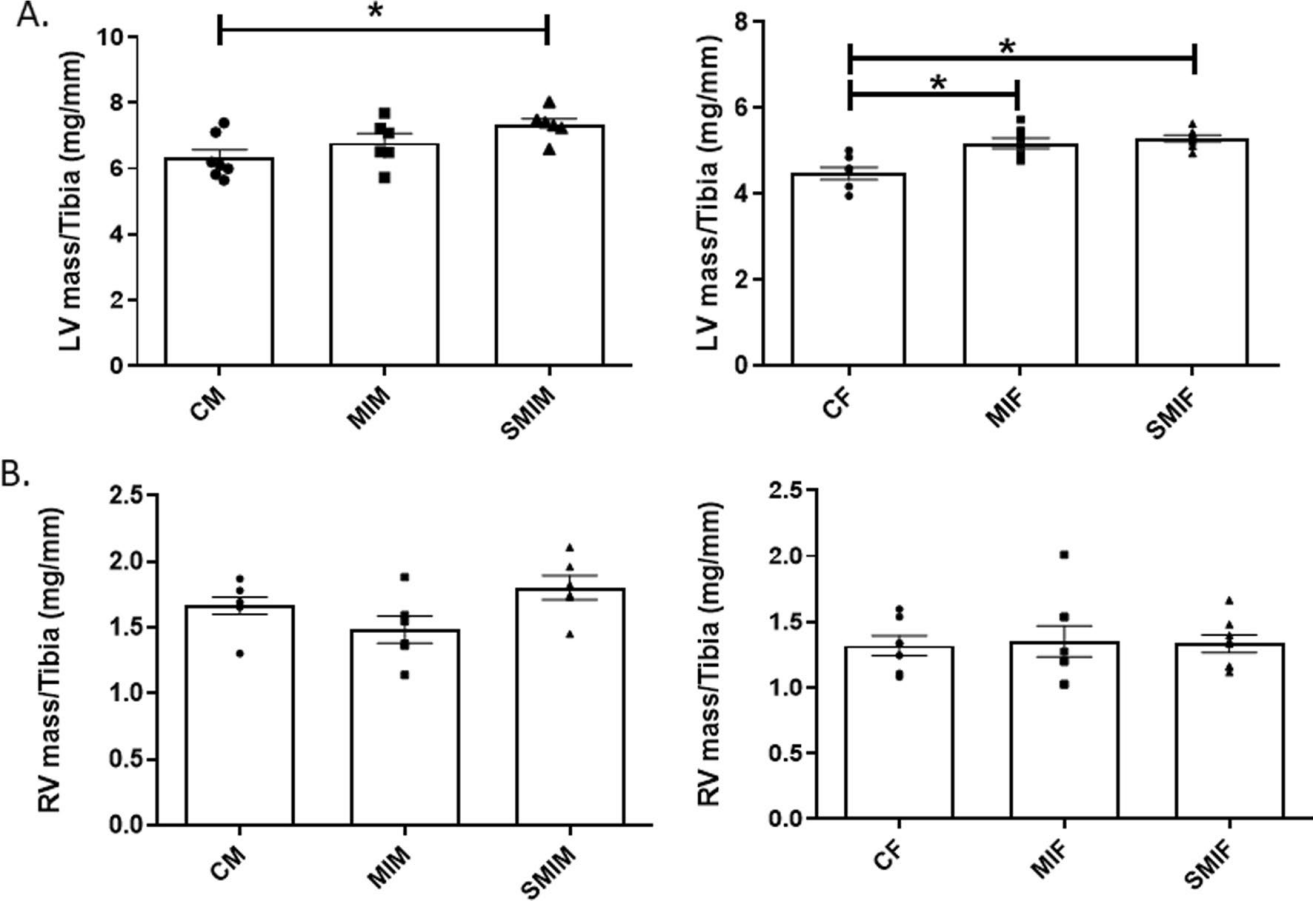
Effect of myocardial infarction (MI) and cigarette smoke (CS) on left ventricular (LV) and right ventricular (RV) mass in males and females. **A** Smoke exposure did not have a significant effect on LV mass of both sexes post-MI. In female groups, LV mass significantly increased post-MI regardless of smoke exposure. However, in male groups, only CS-exposed MI mice showed significantly increased LV mass. **B** Although smoking male mice had a greater RV mass on average post-MI, none of the four groups showed significant RV mass increase. *N* = 7 (CF), 7 (CM), 8 (MIF), 6 (MIM), 8 (SMIF), and 6 (SMIM). *CM/CF* control male/female, *MI* myocardial infarction, *MIF* MI female group, *MIM* MI male group, *SMIF* smoking MI female group, *SMIM* smoking MI male group. *p* ≤ 0.05 (*); ANOVA

Increased LV mass is associated with high cardiovascular mortality and worse prognosis. LV mass was found to be increased in both male groups post-MI regardless of smoke exposure (6.79 ± 0.28 MIM and 7.33 ± 0.19 SMIM vs. 6.33 ± 0.25 CM), but only SMIM mice showed significant results, *p* ≤ 0.05 (Fig. 5A). Although SMIM had a greater LV mass on average than MIM mice, the difference did not reach a significant level. Both female groups (MIF and SMIF) subjected to MI surgery had greater LV mass than control group, 5.19 ± 0.12 MIF and 5.30 ± 0.07 SMIF vs. 4.48 ± 0.17 CF, *p* ≤ 0.05. As with males, smoke exposure did not make a significant change in LV mass between female groups post-MI (Fig. 5A). Regarding the right ventricle, the SMIM group showed higher RV mass compared to both MIM and control groups, 1.80 ± 0.09 SMIM vs. 1.48 ± 0.10 MIM and 1.67 ± 0.07 CM, but statistical significance was not reached (Fig. 5B). Female mice showed no changes in RV mass among groups: 1.33 ± 0.07 SMIF, 1.35 ± 0.12 MIF, and 1.32 ± 0.09 CF (Fig. 5B).

### CS exposure enhances right ventricular area in smoking MI male mice in the absence of pulmonary congestion

**Fig. 6:**
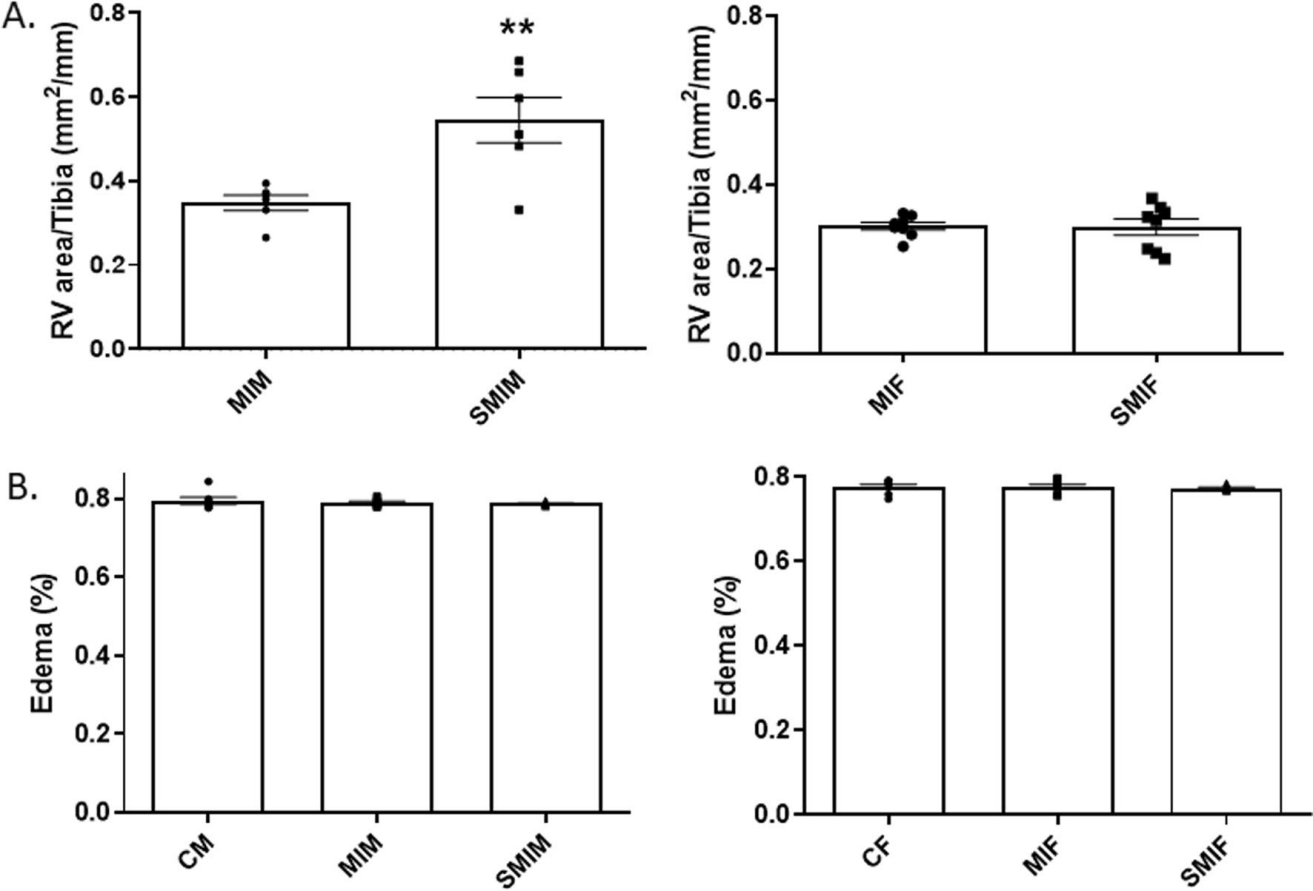
Effect of cigarette smoke (CS) on right ventricular (RV) areas and lung edema in males and females. **A** CS-exposed MI male group had greater RV areas compared to CS-naive MI males. Female groups had comparable RV area post-MI irrespective of smoke exposure. **B** No group developed significant pulmonary fluid accumulation. *N* = 7 (CF), 7 (CM), 8 (MIF), 6(MIM), 8 (SMIF), and 6 (SMIM). *CM/CF* control male/female, *MI* myocardial infarction, *MIF* MI female group, *MIM* MI male group, *SMIF* smoking plus MI female group, *SMIM* smoking plus MI male group. *p* ≤ 0.01 (**); *t* test (**A**) and ANOVA (**B**)

SMIM mice showed significantly larger RV areas compared to MIM mice and control counterparts, 0.54 ± 0.05 vs. 0.35 ± 0.02, *p* < 0.01 (Fig. 6A). Female groups showed no significant changes in RV areas post-MI regardless of smoke exposure, 0.30 ± 0.02 SMIF vs. 0.30 ± 0.01 MIF (Fig. 6A). Lung edema develops as a result of increased pressure in the LV due to the occurrence of systolic and diastolic dysfunction post-MI. Although SMIM mice were prone to have higher fluid percentage in lungs after MI and in consideration of the observed LV remodeling within this group, none of the male (0.80 ± 0.01 CM, 0.79 ± 0.00 MIM, and 0.79 ± 0.00 SMIM) nor female (0.78 ± 0.01 CF, 0.78 ± 0.00 MIF, and 0.77 ± 0.00 SMIF) groups showed significant differences, which suggests the less likely implication of pulmonary congestion in RV remodeling (Fig. 6B).

### Collagen accumulation was more pronounced in CS exposed male group post-MI

**Fig. 7:**
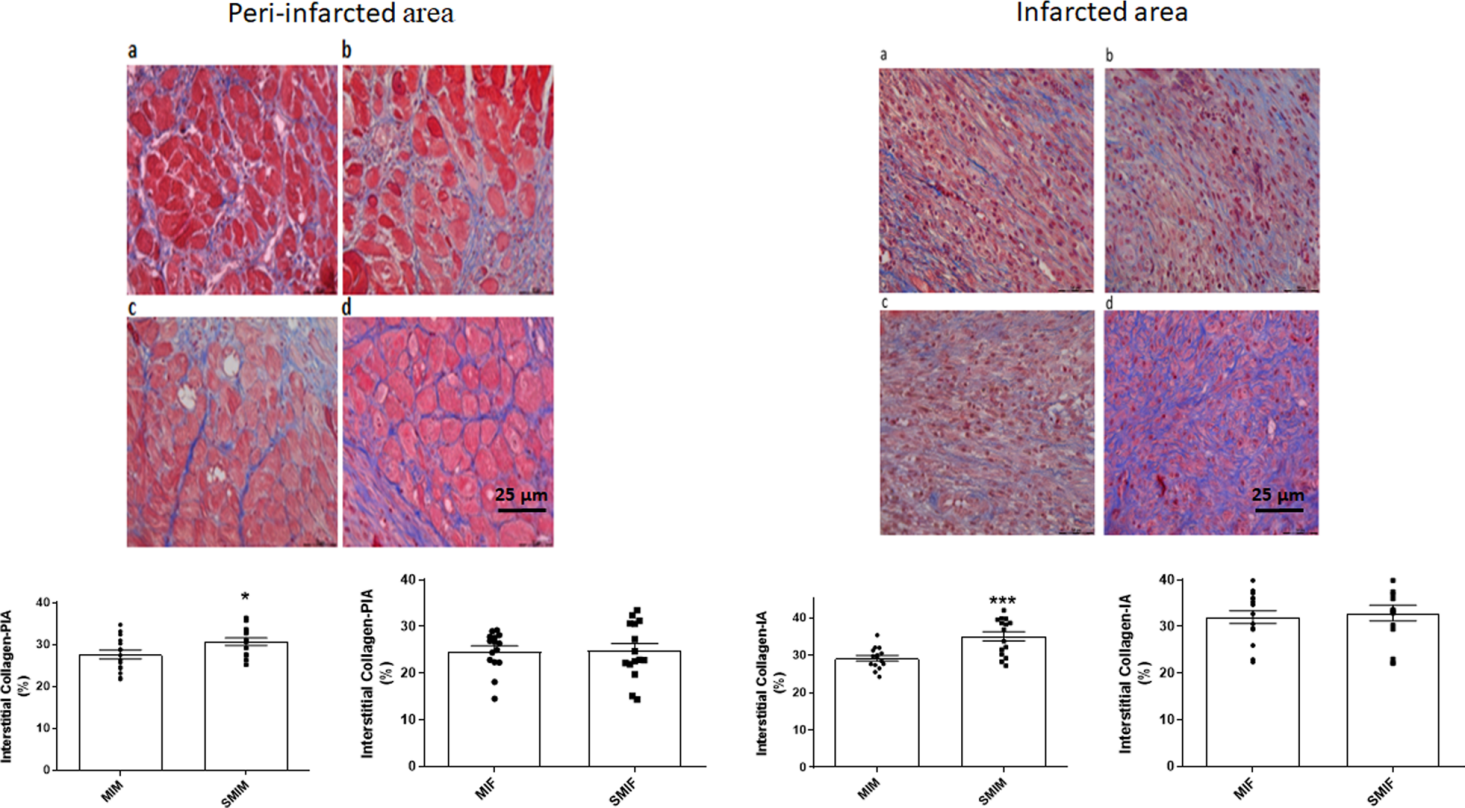
Effect of cigarette smoke (CS) on collagen content of the infarcted and peri-infarcted areas in males and females. Collagen accumulation in both infarcted and peri-infarct area (IA and PIA, respectively) showed significance enhancement in CS-exposed MI male mice only. **a** = MIF (*n* = 15), **b** = MIM (*n* = 15), **c** = SMIF (*n* = 15), and **d** = SMIM (*n* = 15). *MI* myocardial infarction, *MIF* MI female group, *MIM* MI male group, *SMIF* smoking plus MI female group, *SMIM* smoking plus MI male group. *p* ≤ 0.05 (*) and *p* ≤ 0.001 (***); *t* test

SMIM group showed significantly higher collagen accumulation in both infarcted and peri-infarct areas at day 7 post-MI in comparison with MIM mice (35.02 ± 2.01 vs. 29.17 ± 1.17, *p* < 0.001 and 30.70 ± 1.40 vs. 27.63 ± 1.67, *p* < 0.05, respectively). No significant difference was observed between female groups in infarcted (32.90 ± 2.31 SMIF vs. 31.99 ± 1.90 MIF) or peri-infarcted collagen (24.78 ± 2.13 SMIF vs. 24.76 ± 1.47 MIF) (Fig. 7).

### CS exposure enhances left ventricular inflammation in MI males

**Fig. 8:**
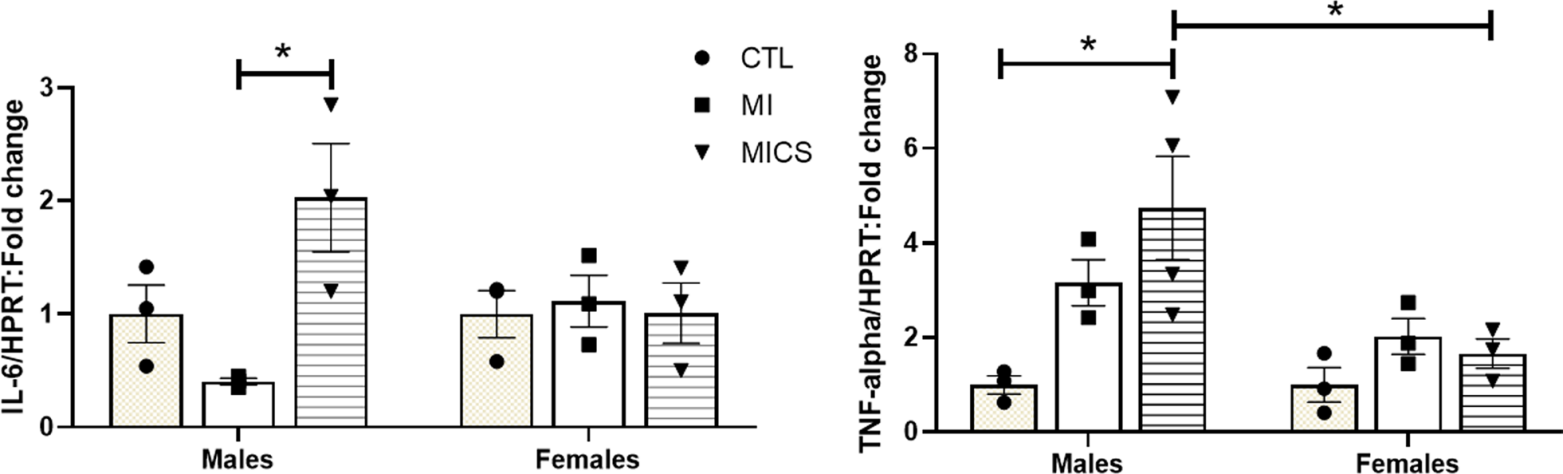
Effect of cigarette smoke (CS) on IL-6 and TNF-α proinflammatory markers fold change at day 7 post-MI. **A** CS-exposed MI male group had greater IL-6 mRNA expression levels compared to CS-naive MI males. Female groups had comparable IL-6 mRNA expression levels post-MI irrespective of smoke exposure. No difference across sexes was observed (**B**) CS-exposed MI male group had greater TNF-α mRNA expression levels compared to control males and CS-exposed MI female group. Female groups had comparable TNF-α mRNA expression levels post-MI irrespective of smoke exposure. *N* = 3 in each group. *CTL* control, *MI* myocardial infarction, *MICS* smoking plus MI. *p < 0.05; ANOVA

Both IL-6 and TNF-α pro-inflammatory mRNA expression increased significantly in the left ventricles of CS exposed MI male mice at day 7 post-MI (Fig. 8). Unlike female groups that showed comparable levels of IL-6 mRNA expression, CS-exposed MI male group had a significantly higher IL-6 mRNA expression at day 7 post-MI when compared with CS-naïve MI male mice (2.03 ± 0.47 vs. 0.40 ± 0.02 *p* < 0.05) (Fig. 8A). No changes were observed with TNF-α mRNA expression among female groups. However, CS-exposed MI male groups exhibited a significant increase in TNF-α mRNA expression when compared to control males and CS-exposed MI female mice (4.74 ± 1.09 vs. 0.99 ± 0.19 and 1.66 ± 0.31, respectively, *p* < 0.05) (Fig. 8B).

## Discussion

The process of LV remodeling after MI is characterized by a series of progressive molecular, cellular, and extracellular matrix (ECM) changes [5, 31]. LV remodeling after permanent LAD leads to infarct expansion, hypertrophy of non-infarcted area, increased collagen accumulation in the infarcted and non-infarcted areas; all together, the changes lead to progressive dilatation and ultimately to impaired LV physiology [31] and eventual progression to chronic heart failure [21]. However, this response is variable between individuals within the same sex and between sexes and can determine the extent of heart affected when it comes to molecular, physiological, and structural changes [32]. Reports from clinical studies suggests that after MI, women develop less adverse LV remodeling than men with better preservation of LV size and function [33, 34], which is widely attributed to their different sex hormones [25, 33, 35, 36]. In animal models of MI, the literature shows inconsistency. While some studies reported worse cardiac remodeling in male than in female rodents [37–40], others did not show any differences between sexes [21, 22, 41]. Since male and female sexes have different LV mass and volume, their response to the same extent of ischemic injury is expected to show discrepancy and lead to confusion in interpretation. Each sex was compared with the same sex, and direct comparison between males and females, except for inflammatory marker fold change, was avoided in the present study.

Previous studies using male rat models of MI showed that smoke exposure is associated with worse cardiac function and/or cardiac dilatation [42–44]. In accordance with these studies, we observed that males exposed to smoke had lower LVEF, more pronounced increases in LVEDV and LVESV than CS-naïve males at day 7 post-MI. The gap between these two groups at day 1 widened at day 7. It is worth mentioning that to eliminate any confounding factor, such as temporal remodeling within the 24 h following MI, study design included only mice with comparable infarct size at day 1. Thus, smoking is the sole factor of discrepancy found in remodeled hearts in male sex at day 7 post-MI. Our observations on EF indicate that smoking is altering the overall contractility independent of infarct size as early as day 1. Interestingly, CS-exposed female groups showed no differences with regard to LVEDV, LVESV and LVEF compared to CS-naïve females either at day 1 or at day 7 post-MI, although LV adverse remodeling was in progress through 7 days. These findings indicate that CS exposure worsens the progression of cardiac dysfunction post-MI in male sex only and in a significant manner.

Since the LV base remains the main part of the heart generating force of contraction, assessment of this region can provide important information. In fact, given the comparable LVEF prior to MI surgery between the male groups regardless of smoke exposure, significantly lower fractional area change of LV base in CS-exposed MI males indicated that CS compromised the compensatory function of the remaining tissue and uncovered the malfunctioning tissue upon facing the hemodynamic stress that existed post-MI. This finding suggested that, alongside promoting inflammation, CS compromises LV function at day 1 and represents a unique and important factor implicated in worsened cardiac remodeling in CS-exposed MI male mice. Distinctively, female groups did not differ in fractional area change of LV base at day 1. The described discrepancy existed at an early stage post-MI between sexes and could be related to discrepancies in contractility or electrophysiology, both of which are well known targets for estrogen [45].

Seven days post-MI, increased LV size is accompanied by increased cross-sectional area of myocytes in both the peri-infarct and remote areas [46–49]. Having greater LV mass at the onset of MI and progressive increase in LV mass post-MI are associated with an increased incidence of adverse clinical outcomes [50, 51]. Our results show that MI male mice manifested an increase in their LV mass, but only CS-exposed ones reached statistical significance at day 7 post-MI compared to control male group. Although smoking infarcted males had greater LV mass than non-smoking ones, this result was not significant. Lack of statistical significance might perhaps be attributed to relatively small number of mice in male groups. As for female sex, LV mass significantly increased in both MI groups irrespective of smoke exposure when compared to control group. Similarly, infarct size is strongly correlated with worse outcomes after transmural myocardial infraction in both sexes [52–54]. At 7 day post-MI, infarct size was significantly high only in CS-exposed MI male mice compared to their CS naïve counterparts. However, no significant difference was observed in female sex at day 7 post-MI_._ This indicates that infarct expansion occurred in males, but not females.

In some previous clinical studies, the smoker showed better prognosis than non-smoker [27, 55, 56]. A plausible explanation of the “smoker’s paradox” is that smokers were on average 14 years younger than non-smokers and had fewer atherosclerotic risk factors and comorbidities [57]. A large study involving pooled analysis of 18 randomized controlled trials with up to 5 year follow-up clearly demonstrated that after multivariable adjustment for potential confounders, smoking is a strong independent predictor of death, cardiac death, MI, stent thrombosis, and target lesion failure [58]. A recent clinical study, however, reported that smoking status has no impact on infarct size, while sex does. Female patients with ST-elevation myocardial infarction showed smaller myocardium at risk, smaller infarct size, and larger myocardial salvage index [59]. Our study differs from the above clinical studies in many ways. First, the mice in our study (irrespective of sex and smoke exposure) were in the same age range and free of comorbidities, unlike clinical studies. Second, we selected mice having comparable infarct size at day 1 to show the impact of smoking throughout 7 days of cardiac remodeling. Third, while the patients in the clinical studies received reperfusion treatment, the mice in our study underwent permeant coronary artery ligation. Altogether, our study revealed that unlike clinical studies, CS status has worse impact on left ventricular remolding post MI, especially in males. Female sex alleviated the worsen impact of CS post-MI, at least for the first 7 days of cardiac remodeling post MI.

ECM composition fractions and percentages are in constant change post-MI with, net ECM accumulation the result of synthesis minus degradation [60, 61]. A good example is the collagen-rich reparative scar that determines an early surge of collagen deposition from around day 5 post-MI to replace the massive loss of cardiomyocytes in the infarcted area [62]. This has been documented experimentally in infarcted rats, in which collagen III gene expression is initiated 2 day post-MI and continues to increase up to 21 day post-MI. However, collagen I gene expression starts to increase 4 day post-MI, peaks at 7 day post-MI, and stabilizes by 21 day post-MI [63]. In our study, CS-exposed MI male mice presented greater collagen accumulation in both infarcted and peri-infarcted areas than CS-naïve MI male mice at day 7 post-MI (Fig. 7). However, an experimental study with a male rat model of MI showed comparable results regarding collagen content in CS-exposed and CS-naïve groups [43]. On the other hand in post-MI female groups no statistically significant differences were seen in interstitial collagen density in both infarcted and peri-infarct area between CS-exposed and CS-naïve females at day 7 post-MI. This might be attributed to the role of estrogen in other organs to suppress collagen accumulation [64, 65]. An in vitro study showed that 17β-estradiol along with its metabolites, as well as progesterone, inhibit cardiac fibroblast growth [66]. This notion has been supported by in vivo studies in which ovariectomized rats with MI showed more intense cardiac collagen accumulation than intact rats with MI [67]. Experimental studies with estrogen receptors also provide further information regarding the impact of estrogen in cardiac fibrosis [68, 69]. The anti-fibrotic property of estrogen was previously associated with its inhibitor action on angiotensin II and endothelin-1, which promote fibrosis in part by inducing TGFβ1 production [70]. This can partially explain the differences in response to CS exposure between MI males and females in our present study.

Pulmonary congestion as a result of LV dysfunction is a common clinical manifestation of dilated LV, which can cause RV dysfunction and remodeling. However, RV function can deteriorate even in the absence of pulmonary hypertension and alteration in RV afterload in case of acute MI involving the left ventricle [71]. Furthermore, it was observed that RV remodeling, even spared from initial ischemic damage, involves cardiac remodeling that is originated from LV post-MI [72–74]. In the present study, we observed significantly increased RV mass only in CS-exposed MI male group compared to CS-naïve MI and control groups. However, CS-exposed MI male mice had significantly larger RV area than CS-naïve MI males, whereas no difference was found between female groups. This finding suggests dilatation of RV rather than hypertrophy, which is usually a consequence of increased pulmonary artery pressure. In line with this, pulmonary congestion did not differ between groups, indicating that development of RV remodeling was independent of pulmonary congestion. Of note, chronic exposure to CS can cause pulmonary vascular resistance and cor pulmonale, which refers to RV enlargement resulting from pulmonary hypertension [75]. Given the duration of CS exposure in the present study, development of cor pulmonale and its implication in RV remodeling are less likely.

CS exacerbates ROS production that overwhelms intracellular antioxidant mechanisms. In addition, tobacco smoke contains substantial amounts of ROS and chemicals that weaken antioxidant defense mechanisms, boost inflammatory response, and worsen damage, both in the presence and/or the absence of cardiac morbidities [7, 76, 77]. Numerous studies in the literature revealed that CS exposure enhances gene expression of pro-inflammatory cytokines in the cardiac tissue and causes systemic inflammation by increasing circulating pro-inflammatory cytokines. In accordance with these observations, we documented a significant increase in the mRNA expression of two major pro-inflammatory markers, IL-6 and TNF-α, at day 7 post-MI in MICS male group only. Both IL-6 and TNF-α spike with initial acute inflammation post-MI (day 1–day 3) and decrease to normal levels during transition to granulation phase post-MI (day 4–day 7) [78]. Persistent inflammation could be the attributing factor to the pronounced cardiac deterioration observed in CS-exposed MI male group that was not seen in the female counterparts. In fact, prolonged inflammation following MI is associated with poor prognosis and a high risk of systolic dysfunction [79]. Estrogen well known anti-inflammatory and antioxidant effects might have played a crucial role in this process by curbing the CS-induced prolonged inflammation in MICS female mice [80, 81]. Further studies are warranted to reach a definitive conclusion around estrogen involvement in this process.

Our study has some limitations. First, we did not follow the functional and structural changes for longer than 7 days after MI. Second, we did not assess the role of sex hormones on the effect of CS on cardiac remodeling post-MI, such as performing ovariectomy or castration, which prevents us from being conclusive regarding underlying mechanisms. Third, it might be argued that exposing animals to CS after MI does not reflect what is happening with patients. Continued smoking following an acute coronary syndrome is associated with greater mortality and patients are recommended to stop smoking [82]. Even with counseling, however, smoking relapse remains a significant issue, especially in Lebanon, with 50–60% of patients with an acute coronary syndrome continuing to smoke after discharge from the hospital [83–85]. Fourth, our study design does not permit an assessment of the effect of smoking per se on cardiac function nor can we completely dismiss the possibility that CS exposure may have induced stress that affected the cardiovascular system; however, in our study we used two different sexes that were subjected to the same stressors and thus the only difference between groups was sex. In addition, we did not observe any gross indications that CS exposure induced stress. Fifth, it is possible that the CS protocol induces stress in mice and consequently has an impact on cardiovascular physiology; however, both sexes were subjected to the same CS protocol. Sixth, ischemia–reperfusion was not performed in our study, but will be done in future experiments; however, a significant percentage of MI patients are not reperfused and suffer from permanent occlusion MIs [86, 87]. Finally, CS exposure was performed for 2 weeks before MI and it would be informative to assess longer exposure times. The key goal, however, was to eliminate the risk of CS-induced cardiomyopathy that normally occur with long-term CS exposure and focus solely on acute events. Nonetheless, our study provides novel data on sex-differences in the response of the infarcted heart to CS.

## Perspectives and significance

Associated with significant morbidity and mortality, MI is the most common cause of death among patients with CVD in industrialized countries [88]. CS is a major cause of most CVD, and especially, coronary heart disease for both men and women [16]. However, the difference in extent of LV damage after MI, has not been fundamentally assessed between smoking men and women before. In the present study, it was shown that CS exposure exacerbated both left and right ventricular remodeling only in males at an early stage of post-MI. Females did not display a significant structural and/or functional alteration within 7 days of cardiac remodeling post-MI upon CS exposure. Worsened RV remodeling in males was independent of pulmonary congestion.

Further studies are also required to illuminate the differences between the sexes and the underlying potential mechanisms. Molecular and cellular time continuum of the different MI response phases should be studied more in greater detail between sexes. Understanding which factors contribute to this discrepancy will provide mechanistic insight into how the progression to heart failure is oriented in both sexes and identify new personalized targets to examine in males and females, separately or both similarly.

## Data Availability

All data generated or analyzed during this study are included in this published article.
